# GROMTools: scalable individual-level GReX imputation for mega-biobank-scale cohorts

**DOI:** 10.1093/bioinformatics/btag719

**Published:** 2026-10-01

**Authors:** Marios Anyfantakis, Sanan Venkatesh, Jamie J R Bennett, Chenyu Liu, Gabriel E Hoffman, Panos Roussos, Georgios Voloudakis

**Affiliations:** Department of Psychiatry, Icahn School of Medicine at Mount Sinai, New York, NY, United States; Center for Disease Neurogenomics, Icahn School of Medicine at Mount Sinai, New York, NY, United States; Friedman Brain Institute, Icahn School of Medicine at Mount Sinai, New York, NY, United States; Department of Genetics and Genomic Sciences, Icahn School of Medicine at Mount Sinai, New York, NY, United States; Mental Illness Research, Education, and Clinical Center (VISN 2 South), James J. Peters VA Medical Center, Bronx, NY, United States; Center for Precision Medicine and Translational Therapeutics, James J. Peters VA Medical Center, Bronx, NY, United States; School of Medicine, University of Crete, Heraklion, Greece; Department of Psychiatry, Icahn School of Medicine at Mount Sinai, New York, NY, United States; Center for Disease Neurogenomics, Icahn School of Medicine at Mount Sinai, New York, NY, United States; Friedman Brain Institute, Icahn School of Medicine at Mount Sinai, New York, NY, United States; Department of Genetics and Genomic Sciences, Icahn School of Medicine at Mount Sinai, New York, NY, United States; Mental Illness Research, Education, and Clinical Center (VISN 2 South), James J. Peters VA Medical Center, Bronx, NY, United States; Center for Precision Medicine and Translational Therapeutics, James J. Peters VA Medical Center, Bronx, NY, United States; Department of Psychiatry, Icahn School of Medicine at Mount Sinai, New York, NY, United States; Center for Disease Neurogenomics, Icahn School of Medicine at Mount Sinai, New York, NY, United States; Friedman Brain Institute, Icahn School of Medicine at Mount Sinai, New York, NY, United States; Department of Genetics and Genomic Sciences, Icahn School of Medicine at Mount Sinai, New York, NY, United States; Mental Illness Research, Education, and Clinical Center (VISN 2 South), James J. Peters VA Medical Center, Bronx, NY, United States; Center for Precision Medicine and Translational Therapeutics, James J. Peters VA Medical Center, Bronx, NY, United States; Department of Psychiatry, Icahn School of Medicine at Mount Sinai, New York, NY, United States; Center for Disease Neurogenomics, Icahn School of Medicine at Mount Sinai, New York, NY, United States; Friedman Brain Institute, Icahn School of Medicine at Mount Sinai, New York, NY, United States; Department of Genetics and Genomic Sciences, Icahn School of Medicine at Mount Sinai, New York, NY, United States; Mental Illness Research, Education, and Clinical Center (VISN 2 South), James J. Peters VA Medical Center, Bronx, NY, United States; Center for Precision Medicine and Translational Therapeutics, James J. Peters VA Medical Center, Bronx, NY, United States; Department of Psychiatry, Icahn School of Medicine at Mount Sinai, New York, NY, United States; Center for Disease Neurogenomics, Icahn School of Medicine at Mount Sinai, New York, NY, United States; Friedman Brain Institute, Icahn School of Medicine at Mount Sinai, New York, NY, United States; Department of Genetics and Genomic Sciences, Icahn School of Medicine at Mount Sinai, New York, NY, United States; Mental Illness Research, Education, and Clinical Center (VISN 2 South), James J. Peters VA Medical Center, Bronx, NY, United States; Center for Precision Medicine and Translational Therapeutics, James J. Peters VA Medical Center, Bronx, NY, United States; Department of Psychiatry, Icahn School of Medicine at Mount Sinai, New York, NY, United States; Center for Disease Neurogenomics, Icahn School of Medicine at Mount Sinai, New York, NY, United States; Friedman Brain Institute, Icahn School of Medicine at Mount Sinai, New York, NY, United States; Department of Genetics and Genomic Sciences, Icahn School of Medicine at Mount Sinai, New York, NY, United States; Mental Illness Research, Education, and Clinical Center (VISN 2 South), James J. Peters VA Medical Center, Bronx, NY, United States; Center for Precision Medicine and Translational Therapeutics, James J. Peters VA Medical Center, Bronx, NY, United States; Department of Psychiatry, Icahn School of Medicine at Mount Sinai, New York, NY, United States; Center for Disease Neurogenomics, Icahn School of Medicine at Mount Sinai, New York, NY, United States; Friedman Brain Institute, Icahn School of Medicine at Mount Sinai, New York, NY, United States; Department of Genetics and Genomic Sciences, Icahn School of Medicine at Mount Sinai, New York, NY, United States; Mental Illness Research, Education, and Clinical Center (VISN 2 South), James J. Peters VA Medical Center, Bronx, NY, United States; Center for Precision Medicine and Translational Therapeutics, James J. Peters VA Medical Center, Bronx, NY, United States; Department of Artificial Intelligence and Human Health, Icahn School of Medicine at Mount Sinai, New York, NY, United States

## Abstract

**Motivation:**

The computational burden of individual-level genetically regulated gene expression (GReX) imputation has risen sharply with the growth of human mega-biobanks and the rapid expansion of transcriptomic imputation models across tissues and single-cell hierarchies. Existing tools were not designed for this setting and require complex, memory-intensive workflows that are poorly matched to shared and cloud-based compute environments, where runtime, memory, and I/O directly determine cost and throughput. GROMTools is an open-source C++ engine with an R interface that exploits sparse prediction weights, streams PLINK2 genotypes, and writes compact binary outputs for scalable individual-level GReX imputation.

**Results:**

In UK Biobank (UKB), benchmarks on chromosome 1 across 50,000–450,000 individuals, 388,017 variants, and 11,724 gene-model pairs from 32 single-nucleus transcriptome imputation models, GROMTools produced near-identical predictions to PrediXcan and PLINK2, with minimum Pearson correlation >0.999 and maximum RMSE <0.001 across all of the imputed genes, while reducing CPU time by about 100-fold and peak memory by about 33-fold. These gains make routine biobank-scale individual-level GReX imputation practical and cost-efficient on standard CPU infrastructure.

**Availability and implementation:**

GROMTools is freely available at https://github.com/voloudakislab/gromtools under the GPL v3 license. Documentation is available at https://voloudakislab.github.io/gromtools/. All scripts used for data quality control and benchmarking, together with log files and a snapshot of the GROMTools code version used for all of our analyses, are archived at DOI: https://doi.org/10.5281/zenodo.21926165.

## 1 Introduction

Transcriptome-wide association studies (TWAS) and related transcriptome-informed analyses have become a central framework in human complex trait genetics. These methods link germline genetic variation to gene expression and downstream phenotypes through genetically regulated gene expression (GReX). Beginning with PrediXcan (Gamazon *et al.* 2015) and FUSION (Gusev *et al.* 2016), they train expression prediction models in reference transcriptome panels and apply the resulting cis-genetic weights to summary- and individual-level biobank genotypes, typically as weighted linear combinations of local variants. This framework enables GReX imputation from genotype data and, in summary-statistics settings, direct gene-trait association testing without matched expression measurements in the target cohort. Unlike summary-statistics workflows such as S-PrediXcan, individual-level GReX imputation generates participant-specific estimates directly from genotype data for use in downstream analyses. Across a wide range of complex traits and diseases, these approaches have yielded reproducible and biologically interpretable findings (Huckins *et al.* 2020; Voloudakis *et al.* 2022; Venkatesh *et al.* 2024).

Yet the scale of individual-level GReX imputation has increased substantially. Large human biobanks now include millions of genotyped participants, while reference prediction models continue to expand across tissues (Gamazon *et al.* 2015; Zhang *et al.* 2019) and increasingly fine single-cell hierarchies (Venkatesh *et al.* 2024; Zeng *et al.* 2024). Consequently, individual-level GReX imputation now increasingly requires aggregating sparse genetic weights across thousands of genes, many tissue and cell-type models, and very large cohorts. Existing tools were not designed for this setting and therefore depend on complex, memory-intensive workflows deployed in high-performance or cloud computing environments. In shared and fee-for-service settings, runtime, memory use, and I/O thus directly affect both cost and throughput (Krissaane *et al.* 2020; McDonald *et al.* 2012).

To address this individual-level GReX imputation bottleneck, we developed GROMTools (Genetically Regulated Omics Tools), an open-source GReX imputation engine implemented in C++ with an R interface. GROMTools exploits sparse prediction weights, streams PLINK2 genotype data efficiently, and writes compact binary outputs, enabling scalable individual-level GReX imputation without proportionate increases in memory usage. Using incremental subsampling of the UK Biobank (Sudlow *et al.* 2015), we benchmarked GROMTools against PrediXcan and PLINK2 (Chang *et al.* 2015) and observed concordant GReX predictions together with substantial gains in CPU runtime and memory efficiency.

## 2 Methods

### 2.1 Prediction framework and input preparation

GReX is a large sparse linear prediction problem. Let Y=G×B, where $G\in R^{n\times m}$ is the genotype matrix for n individuals and m variants, $B\;\in R^{m\times k}$ is the sparse matrix of m model-specific variant weights across k gene-model pairs, and $Y\in R^{n\times k}$ is the resulting matrix of individual-level imputed expression values. At biobank scale, the main challenge is computational rather than statistical: naive implementations typically load large genotype matrices into memory and apply general dense matrix operations that do not exploit the sparsity of B. GROMTools preserves the standard linear GReX model, but improves its implementation by streaming genotype data in SNP chunks and restricting computation to nonzero weights, thereby reducing memory usage, avoiding repeated scans of large genotype files, and accelerating matrix multiplication.

Before computation, the current workflow requires a prior QC step in which weight-file predictors are matched to genotype variants using chromosome-position-reference-alternate (CPRA), with complementary-allele matches permitted and effect-allele alignment resolved; full details are described elsewhere (Venkatesh *et al.* 2024) and we provide a companion repository (voloudakislab/gromtools_QC_helper) that implements this approach. Following this QC step, GROMTools maps genotype variants from the .PVAR manifest to the corresponding weight-file predictors for imputation. This pre-indexing removes repeated parsing and lookup from the inner computation loop. To reduce genotype access overhead, GROMTools streams PLINK2 binary files (pgen/psam/pvar) directly through the vendored pgenlib library. Genotypes are read in SNP chunks rather than loading the full genotype matrix into memory, and optional sample subsetting is applied before computation so that downstream buffers are sized only to the analyzed individuals. Together, these design choices reduce both memory use and input overhead, improving scalability to large cohorts.

Prediction weights are supplied as tab-separated files, one per transcriptomic imputation model, containing variant identifiers, effect alleles, and weight coefficients. At startup, GROMTools loads all weights and organizes them into a unified sparse representation indexed by variant. Because the full weight matrix spanning *M* variants and *K* gene-model pairs is highly sparse, this representation remains compact and allows each variant to traverse only the gene-model pairs with nonzero weights, avoiding unnecessary scans over unrelated predictors.

### 2.2 Computational strategy and memory management

The core computation is implemented in C++ as sparse linear updates over the weight structure, accumulating GReX predictions blockwise across samples. For each variant, only the connected gene-model pairs are visited, and the corresponding prediction vectors are updated using the C-interface to the Basic Linear Algebra Subprograms (BLAS) library for efficient dense accumulation. To improve throughput, updates are further decomposed into row chunks across samples, allowing computation to proceed over contiguous slices of the genotype and output vectors in a cache-friendly manner. Intel Math Kernel Library (MKL) can also be used as an alternative backend where available. In the benchmarks reported here, MKL was not enabled for GROMTools, so as to provide a conservative estimate of performance under a standard non-optimized BLAS configuration.

To control memory usage, GROMTools avoids materializing the full output matrix in memory. Instead, it allocates dense output columns only when a gene-model pair is first touched during computation, accumulates contributions while that column remains active, and flushes completed columns to disk once all contributing variants have been processed. Export is performed in batches to a preallocated .grom file in contiguous column-major layout. As a result, peak memory depends primarily on the number of simultaneously active output columns, together with the current genotype chunk, rather than on the full cohort-by-gene matrix, thereby improving scalability to very large cohorts on standard CPU nodes.

### 2.3 Software implementation and user interface

GROMTools is implemented in C++17 and exposed through an R interface using Rcpp. Genotype I/O uses vendored PLINK2/pgenlib code through RPgenReader.cpp, so an external pgenlib library is not required. The package is built with a standard R package toolchain using configure and Makevars. A pre-built Docker container image (https://hub.docker.com/r/marvany/ubuntu-r422-plink2) is provided for reproducible execution.

GROMTools exposes two user-facing wrapper functions, grom_impute() and grom_read(). In its standard use case, grom_impute() receives as input a tab-delimited weights table (weights_path) containing the columns chromosome, ancestry, model_ID, gene, rsid, and weight, together with pgen_dir, the directory containing the corresponding PLINK2 genotype files (.pgen, .pvar, .psam). The chromosome field in the weights table is expected to use the chr* convention (e.g. chr1), which is used to map each set of gene-model weights to the corresponding chromosome-specific PLINK2 file. The genotype files are therefore likewise expected to be chromosome split and contain the literal chr* string in their filenames. To prevent erroneous chromosome assignment, GROMTools performs internal checks to ensure exact chromosome matching and to avoid ambiguous mappings such as chr1 being incorrectly assigned to chr11. Additional quality-control procedures are applied throughout the mapping process, and the resulting mappings to the .pvar and .pgen files are written to the meta/ directory for optional user inspection. The grom_impute() function does not impose a minor-allele-frequency threshold and can incorporate rare variants when they are present in both the prediction model and the target genotype data and pass variant-matching and quality-control procedures.

The function further receives an input string (grom_pfx), which specifies the prefix of the output files. These outputs include grom_pfx.grom, a binary matrix containing the imputed gene-expression values for all model-gene pairs across all chromosomes; grom_pfx.gid, which stores the mapping of output columns to model-gene identifiers and serves a role analogous to the .pvar file in PLINK2; and grom_pfx.sid, which stores the mapping of output rows to samples and is analogous to the .psam file.

The companion function, grom_read(), provides access to previously generated GROMTools output and takes grom_pfx as its primary input. It also exposes optional arguments for selective loading of models, genes, or individuals, enabling targeted access to subsets of the imputed matrix without requiring full import of all results.

All remaining arguments of grom_impute() and grom_read() are optional. Detailed descriptions of these parameters, together with usage examples, are provided in the package documentation and in the vignettes/getting-started.md available in the GitHub repository.

### 2.4 Benchmark design and evaluation

We benchmarked GROMTools against PrediXcan and PLINK2 --score using UK Biobank genotype data. To assess scalability, all methods were evaluated at sample sizes of 50,000, 100,000, 150,000, 250,000, 350,000, and 450,000 individuals. Benchmarking was performed on chromosome 1, comprising 388,017 variants and 11,724 gene-model pairs from 32 transcriptome imputation models (Table S1, available as supplementary data at *Bioinformatics* online), obtained from our recent work (Venkatesh, 2026; Venkatesh *et al.* 2024). For PrediXcan, all method-specific preprocessing was completed before the timed scoring run and was excluded from the reported runtime. Specifically, PLINK 2 genotype files (.pgen/.pvar/.psam) were converted to bgzipped VCF files using plink2 --export vcf bgz and indexed with Tabix. Allele-frequency files were generated, region-specific VCF shards were constructed and mean-imputed, and the resulting shards were indexed and merged before running Predict.py. The PrediXcan model SQLite databases were pre-existing model inputs and did not need to be generated for the benchmark. Thus, the reported PrediXcan runtime reflects the scoring step performed by Predict.py, rather than the preceding format conversion, indexing, sharding, or imputation steps. For PLINK 2, the only method-specific preprocessing step was to prepare the score lists, which generated PLINK 2-compatible score files from the prediction models. This step was completed before benchmarking and excluded from the reported runtime. No additional genotype conversion, indexing, sharding, or imputation was performed before the timed PLINK 2 scoring run. For GROMTools, the construction of the required index triplet was included in the reported runtime since its computational cost was negligible. Thus, unlike PrediXcan and PLINK 2, the reported GROMTools runtime included its method-specific preprocessing step. No method benefited from data cached by a preceding run, because each benchmark was conducted under cold-cache conditions. All jobs were run on the Mount Sinai Minerva high-performance computing environment under IBM Spectrum LSF using CPU-only, single-core execution and the same containerized software environment which included all software and dependencies required to run each of the benchmarked methods (https://hub.docker.com/r/marvany/ubuntu-r422-plink2). All methods accessed identical genotype inputs from the same shared filesystem and used the same storage environment for intermediate and output files. To ensure a conservative comparison, GROMTools was run on nodes rated by the LSF scheduler as equivalent to or slower than those used for the comparator methods. Detailed node specifications are provided in Table S2, available as supplementary data at *Bioinformatics* online.

Output concordance between GROMTools and each reference method was assessed by sampling predicted expression values for different sets of 10,000 individuals per gene-model pair and performing pairwise comparisons across methods. For each comparison, we calculated Pearson correlation and root mean square error (RMSE) across all gene-model pairs and reported the minimum Pearson correlation and maximum RMSE observed. Because the methods are deterministic, any residual discrepancies are attributable to minor floating-point rounding differences. Computational performance was evaluated using total computational hours and peak random-access memory (RAM) usage as reported by the LSF scheduler.

## 3 Results

### 3.1 GROMTools preserves GReX estimates

Across all models for our GROMTools predictions, minimum Pearson’s correlation was greater than 0.999 and maximum RMSE was less than 0.001 when compared to both PrediXcan and PLINK2 GReX predictions.

### 3.2 Reduced computational demands enable biobank-scale GReX imputation

Across the different sample subsets, when using GROMTools CPU time and peak memory usage decrease by ∼99% (107-fold against PrediXcan and 165-fold against PLINK2) and ∼96% (∼33-fold), respectively, when compared to either PrediXcan or PLINK2 (Fig. 1) on average across sample sizes (Table S3, available as supplementary data at *Bioinformatics* online, summarizes these performance metrics for each sample subset across methods).

**Figure 1:**
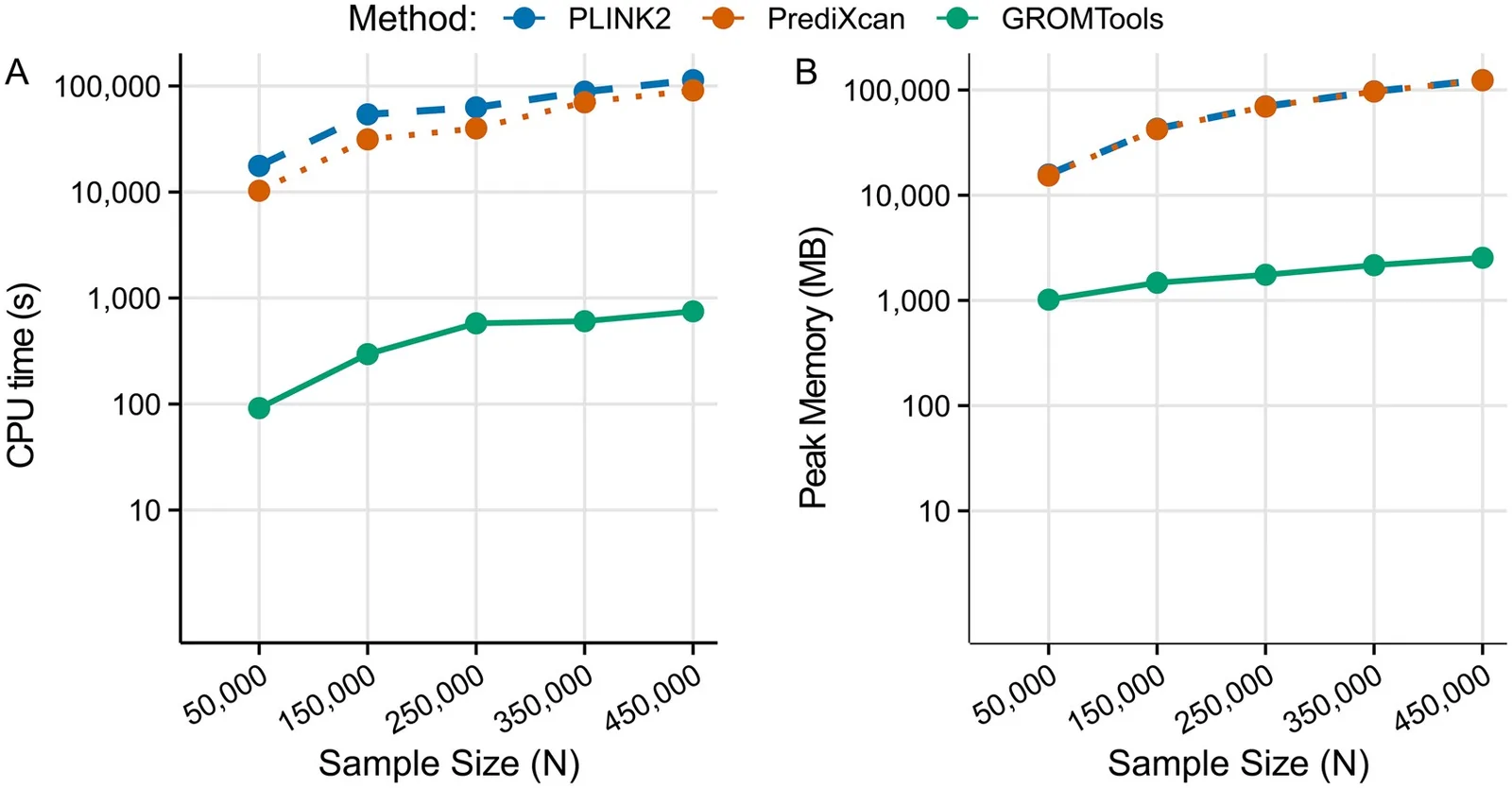
Scaling of CPU time and peak memory usage with sample size. All benchmarks were performed in single-threaded mode. (A) Total CPU time (seconds; log10 scaling) for GROMTools, PLINK2, and PrediXcan across sample sizes from 50,000 to 450,000 individuals for chromosome 1. GROMTools consistently required less CPU time than both comparator methods, with CPU-time reductions ranging from 108.93-fold to 208.75-fold relative to PLINK2 and from 69.02-fold to 121.39-fold relative to PrediXcan. (B) Peak memory usage (MB; log10 scaling) across the same sample sizes for chromosome 1. GROMTools maintained a smaller peak memory footprint, with peak memory reductions ranging from 15.46-fold to 48.82-fold relative to PLINK2 and from 15.13-fold to 48.63-fold relative to PrediXcan. These results highlight the substantially lower computational burden of GROMTools and its improved scalability for biobank-scale GReX imputation.

grom_impute’s binary output format reduces I/O overhead relative to the text-based outputs produced by comparator methods. Across benchmark sample sizes, GROMTools demonstrated average read-character count (rchar) reduced by approximately 32-fold versus PLINK2 and 13-fold versus PrediXcan, and average write-character count (wchar) reduced by approximately 6-fold and 3-fold, respectively (Table S4, available as supplementary data at *Bioinformatics* online). For large numbers of genes or models, comparator methods’ workflows can generate hundreds of gigabytes of output, often requiring chunked execution and subsequent merging or format-conversion steps. The binary outputs can be read directly into downstream analyses in R, reducing storage demands and simplifying biobank-scale individual-level GReX workflows.

## 4 Conclusions

GROMTools addresses a practical bottleneck in individual-level GReX imputation by reducing computational cost and memory requirements while preserving near-identical numerical results relative to baseline workflows. Across UK Biobank-scale benchmarks, in our single-threaded comparisons GROMTools reduces CPU-hours and peak memory by 99% (>100-fold) and 96% (∼33-fold), respectively, supporting routine large-scale GReX estimation on standard CPU-based compute environments. These gains were accompanied by lower I/O burden, as GROMTools streams directly from PLINK files and avoids large text-based score outputs, instead generating a compact binary format that can be streamed directly in R or loaded in chunks for downstream analyses.

GROMTools also demonstrated strong multithreaded scaling. For chromosome 1 GReX imputation in 450,000 UK Biobank participants, parallel efficiency was approximately 100%, 100%, 80%, and 50% using 2, 4, 8, and 16 OpenMP threads, respectively, while peak memory remained below 3 GB and steady across all runs (Table S5, available as supplementary data at *Bioinformatics* online). Stage-specific profiling showed near-linear scaling of the numerical computation, from 327.6 seconds with one core to 20.5 seconds with 16 OpenMP threads. In contrast, PGEN input processing showed diminishing returns beyond four OpenMP threads. Using 16 OpenMP threads, the end-to-end walltime for genome-wide imputation over 1,663,362 variants and 450,000 participants across chromosomes 1 through 22 was 17.4 minutes, with peak memory below 4 GB (Table S6, available as supplementary data at *Bioinformatics* online).

Beyond computational efficiency, GROMTools simplifies workflow execution and, although it internally uses tabular files to improve readability and facilitate modification of transcriptomic imputation models, it also supports importing commonly used database formats such as the standard SQLite .db models established by PrediXcan. The method also produces a single unified output across all evaluated models, avoiding the chunking and post hoc merge steps often required in conventional high-dimensional imputation pipelines, where intermediate outputs can reach hundreds of gigabytes. Together, these features reduce pipeline complexity and improve usability in transcriptome-informed analyses.

The principal limitation of GROMTools is its current lack of internal allele alignment between genotype data and prediction-model weights. Consequently, inputs must be pre-harmonized so that each model effect allele matches the corresponding dosage-counted allele. Unresolved or incorrectly aligned alleles may produce erroneous GReX estimates; recommended harmonization and quality-control procedures are described elsewhere (Zhao *et al.* 2024).

## Supplementary Material

btag719_Supplementary_Data

## Data Availability

UK Biobank genotype data are available to approved researchers subject to UK Biobank access requirements (https://www.ukbiobank.ac.uk/use-our-data/apply-for-access/). The PsychAD prediction models are available through Synapse (https://doi.org/10.7303/syn63181047). Benchmarking results are provided in the Supplementary material. Benchmarking scripts, execution logs, and the GROMTools code snapshot are archived at https://doi.org/10.5281/zenodo.19547332. GROMTools source code is available at https://github.com/voloudakislab/gromtools.
