# Supplementary material for: GROMTools: scalable individual-level GReX imputation for mega-biobank-scale cohorts
Source: Bioinformatics. 2026 Oct 1;42(10):btag719. doi: 10.1093/bioinformatics/btag719 (PMC13680095; doi:10.1093/bioinformatics/btag719)
Supplement: Supplementary file 1 [file btag719_supplementary_data.docx]

Supplementary Info

Supplementary Material

GROMTools: scalable individual-level GReX imputation for mega-biobank-scale cohorts

Authors: Marios Anyfantakis^1-7^, Sanan Venkatesh^1-6^, Jamie Bennett^1-6^, Gabriel E. Hoffman^1-6^, Panos Roussos^1-6^, Georgios Voloudakis^1-6,8^

Affiliations:

^1^Department of Psychiatry, Icahn School of Medicine at Mount Sinai, New York, NY, USA

^2^Center for Disease Neurogenomics, Icahn School of Medicine at Mount Sinai, New York, NY, USA

^3^Friedman Brain Institute, Icahn School of Medicine at Mount Sinai, New York, NY, USA

^4^Department of Genetics and Genomic Sciences, Icahn School of Medicine at Mount Sinai, New York, NY, USA

^5^Mental Illness Research, Education, and Clinical Center (VISN 2 South), James J. Peters VA Medical Center, Bronx, NY, USA

^6^Center for Precision Medicine and Translational Therapeutics, James J. Peters VA Medical Center, Bronx, NY, USA.

^7^University of Crete School of Medicine, University Hospital of Heraklion, Heraklion, Crete, Greece

^8^Department of Artificial Intelligence and Human Health, Icahn School of Medicine at Mount Sinai, New York, NY, USA

**SUPPLEMENTARY TABLES	3**

Supplementary Table 1	3

Supplementary Table 2	4

Supplementary Table 3	5

Supplementary Table 4	5

Supplementary Table 5	6

Supplementary Table 6	6

# SUPPLEMENTARY TABLES

## Supplementary Table 1

**Model**

Excitatory Neuron

Inhibitory Neuron

Immune Cell

Mural Cell

Endothelial Cell

Astrocyte

Oligodendrocyte

Oligodendrocyte Progenitor Cell

Layer 2-3 Intratelencephalic Excitatory Neuron

Layer 3-5 Intratelencephalic Excitatory Neuron 1

Layer 3-5 Intratelencephalic Excitatory Neuron 2

Layer 3-5 Intratelencephalic Excitatory Neuron 3

Layer 5-6 Near-Projecting Excitatory Neuron

Layer 5 Extratelencephalic Excitatory Neuron

Layer 6 Corticothalamic Excitatory Neuron

Layer 6 Intratelencephalic Excitatory Neuron 1

Layer 6 Intratelencephalic Excitatory Neuron 2

Layer 6B Excitatory Neuron

ADARB2 Inhibitory Neuron

Ivy cell (LAMP5 LHX6 Inhibitory Neuron)

Neurogliaform cell (LAMP5 RELN Inhibitory Neuron)

Basket Cell (PVALB Inhibitory Neuron)

Chandelier Cell (PVALB CHC Inhibitory Neuron)

Martinotti and Non-Martinotti cell (SST Inhibitory Neuron)

VIP Inhibitory Neuron

Microglia

Perivascular Macrophage

Adaptive Immune Cell

Pericyte

Smooth Muscle Cell

Vascular Leptomeningeal Cell

Excitatory Neuron

Inhibitory Neuron

**Supplementary Table 1.** Transcriptomic imputation models included in the benchmarking analyses. Only European ancestry models were utilized. Source: Venkatesh et al., 2026.

## Supplementary Table 2

| **Method** | **Sample Size** | **LSF Node** | **Node performance designation** | **Node Type** | **CPU model** | **cpuf** |
| --- | --- | --- | --- | --- | --- | --- |
| **GROMTools** | 50,000 | lh06c31 | Slow | X86_64 | INTELSL24B | 29 |
| **GROMTools** | 100,000 | lh06c16 | Slow | X86_64 | INTELSL24B | 29 |
| **GROMTools** | 150,000 | lh06c15 | Slow | X86_64 | INTELSL24B | 29 |
| **GROMTools** | 250,000 | lh06c31 | Slow | X86_64 | INTELSL24B | 29 |
| **GROMTools** | 350,000 | lh05g27 | Medium | X86_64 | INTELICEL32 | 36 |
| **GROMTools** | 450,000 | lh05g27 | Medium | X86_64 | INTELICEL32 | 36 |
| **PLINK2** | 50,000 | lh05g23 | Medium | X86_64 | INTELICEL32 | 36 |
| **PLINK2** | 100,000 | lh05g07 | Medium | X86_64 | INTELICEL32 | 36 |
| **PLINK2** | 150,000 | lh06c31 | Slow | X86_64 | INTELSL24B | 29 |
| **PLINK2** | 250,000 | lc07e27 | Fast | X86_64 | INTELER23 | 50 |
| **PLINK2** | 350,000 | lc06e60 | Fast | X86_64 | INTELER23 | 50 |
| **PLINK2** | 450,000 | lc06e37 | Fast | X86_64 | INTELER23 | 50 |
| **PrediXcan** | 50,000 | lh06c33 | Slow | X86_64 | INTELSL24B | 29 |
| **PrediXcan** | 100,000 | lh06c20 | Slow | X86_64 | INTELSL24B | 29 |
| **PrediXcan** | 150,000 | lh05g27 | Medium | X86_64 | INTELICEL32 | 36 |
| **PrediXcan** | 250,000 | lc07e11 | Fast | X86_64 | INTELER23 | 50 |
| **PrediXcan** | 350,000 | lh05g21 | Medium | X86_64 | INTELICEL32 | 36 |

**Supplementary Table 2. Compute-node characteristics for individual benchmark runs.** Compute-node characteristics for individual benchmark runs. Each row reports the execution environment for one method-sample-size benchmark. The hostname recorded in each job log was matched against the Minerva LSF host inventory obtained using lshosts -w to determine the node architecture, LSF processor-model class, and CPU factor (cpuf). INTELSL24B, INTELICEL32, and INTELER23 denote dual-socket Minerva nodes equipped with Intel Xeon Platinum 8268 (Cascade Lake), Intel Xeon Platinum 8358 (Ice Lake), and Intel Xeon Platinum 8568Y+ (Emerald Rapids) processors, respectively. For readability, these node classes were designated Slow, Medium, and Fast according to their increasing LSF CPU factors of 29, 36, and 50, respectively. These designations represent relative scheduler ratings and should not be interpreted as processor clock speeds or measured benchmark performance. Compute nodes were allocated by the LSF scheduler according to availability and were not selected for individual methods. CPU information for the PrediXcan benchmark at a sample size of 450,000 was unavailable because node lh06c09 was not returned by the lshosts -w command.

## Supplementary Table 3

| **Method** | **Sample size** | **CPU time (sec)** | **Peak memory (MB)** |
| --- | --- | --- | --- |
| **GROMTools** | 50,000 | 91.53 | 1,017 |
| **GROMTools** | 100,000 | 168.90 | 1,209 |
| **GROMTools** | 150,000 | 295.58 | 1,472 |
| **GROMTools** | 250,000 | 577.11 | 1,753 |
| **GROMTools** | 350,000 | 604.49 | 2,163 |
| **GROMTools** | 450,000 | 748.37 | 2,543 |
| **PLINK2** | 50,000 | 17,648.17 | 15,720 |
| **PLINK2** | 100,000 | 35,257.94 | 29,267 |
| **PLINK2** | 150,000 | 54,028.79 | 42,829 |
| **PLINK2** | 250,000 | 62,862.50 | 69,936 |
| **PLINK2** | 350,000 | 88,437.69 | 97,046 |
| **PLINK2** | 450,000 | 113,614.30 | 124,144 |
| **PrediXcan** | 50,000 | 10,275.93 | 15,383 |
| **PrediXcan** | 100,000 | 20,186.21 | 28,912 |
| **PrediXcan** | 150,000 | 31,367.30 | 42,458 |
| **PrediXcan** | 250,000 | 39,831.50 | 69,542 |
| **PrediXcan** | 350,000 | 70,195.68 | 96,611 |
| **PrediXcan** | 450,000 | 90,841.42 | 123,656 |

**Supplementary Table 3.** Comparison of CPU time and peak memory usage across benchmark sample sizes for GROMTools, PLINK2, and PrediXcan.

## Supplementary Table 4

| **Method** | **Sample Size** | **Read-like I/O bytes (rchar)** | **Write-like I/O bytes (wchar)** |
| --- | --- | --- | --- |
| **GROMTools** | 50,000 | 8.80E+08 | 4.70E+09 |
| **GROMTools** | 100,000 | 1.75E+09 | 9.39E+09 |
| **GROMTools** | 150,000 | 2.62E+09 | 1.41E+10 |
| **PLINK2** | 50,000 | 3.16E+10 | 2.99E+10 |
| **PLINK2** | 100,000 | 5.54E+10 | 5.63E+10 |
| **PLINK2** | 150,000 | 7.92E+10 | 8.28E+10 |
| **PrediXcan** | 50,000 | 1.27E+10 | 1.37E+10 |
| **PrediXcan** | 100,000 | 2.43E+10 | 2.75E+10 |
| **PrediXcan** | 150,000 | 3.59E+10 | 4.12E+10 |

**Supplementary Table 4.** Read-like and write-like I/O volume across benchmark sample sizes for GROMTools, PLINK2, and PrediXcan.

## Supplementary Table 5

| **OpenMP threads** | **Wall time (s)** | **Speedup** | **Parallel efficiency (%)** | **LSF node** |
| --- | --- | --- | --- | --- |
| **1** | 572.0 | N/A | N/A | lh06c32 |
| **2** | 279.0 | 2.1 | 102.5 | 2*lh06c32 |
| **4** | 127.0 | 4.5 | 112.6 | 4*lh06c25 |
| **8** | 90.0 | 6.4 | 79.4 | 8*lh06c25 |
| **16** | 72.0 | 7.9 | 49.7 | 16*lh06c17 |

**Supplementary Table 5.** Multithreaded scaling of GROMTools for chromosome 1 GReX imputation in 450,000 UK Biobank participants using 1-16 OpenMP threads.

## Supplementary Table 6

| **Chromosome** | **Total variant count** | **Engine runtime (s)** |
| --- | --- | --- |
| **1** | 142,709 | 61.6 |
| **2** | 135,015 | 66.5 |
| **3** | 117,966 | 57.9 |
| **4** | 105,855 | 52.1 |
| **5** | 104,121 | 66.6 |
| **6** | 102,252 | 41.0 |
| **7** | 92,578 | 41.2 |
| **8** | 60,673 | 28.5 |
| **9** | 68,136 | 38.5 |
| **10** | 87,739 | 72.7 |
| **11** | 85,221 | 44.9 |
| **12** | 90,537 | 42.7 |
| **13** | 51,344 | 41.7 |
| **14** | 57,432 | 40.6 |
| **15** | 56,819 | 54.2 |
| **16** | 51,874 | 32.2 |
| **17** | 59,054 | 34.5 |
| **18** | 43,543 | 31.4 |
| **19** | 58,677 | 40.1 |
| **20** | 38,405 | 16.5 |
| **21** | 22,922 | 22.3 |
| **22** | 30,490 | 32.5 |
| **All** | 1,663,362 | 960.1 |

**Supplementary Table 6.** Chromosome-specific variant counts and engine runtimes for genome-wide GROMTools GReX imputation in 450,000 UK Biobank participants using 16 OpenMP threads.
